# Optimization of a peptide extraction and LC–MS protocol for quantitative analysis of antimicrobial peptides

**DOI:** 10.4155/fsoa-2018-0073

**Published:** 2018-10-17

**Authors:** Wen Chen, Yoon Y Hwang, Jeremy W Gleaton, James K Titus, Nicholas J Hamlin

**Affiliations:** 1 Craniofacial Health & Restorative Medicine, Naval Medical Research Unit San Antonio, 3650 Chambers Pass BLDG 3610, JBSA Fort Sam Houston, TX, 78234, USA

**Keywords:** antimicrobial peptide, identification and quantification, liquid chromatography, mass spectrometry, peptide extraction

## Abstract

We optimized a peptide extraction and LC–MS protocol for identification and quantification of antimicrobial peptides (AMPs) in biological samples. Amphipathic AMPs were extracted with various concentrations of ethanol, methanol, acetonitrile, formic acid, acetic acid or trichloroacetic acid in water. Yields were significantly greater for extraction with 66.7% ethanol than other extraction methods. Liquid chromatography was accomplished on a C18 column with a linear gradient of acetonitrile–formic acid, and mass spectrometry detection was performed in the positive electrospray multiple reaction monitoring mode by monitoring the transitions at *m/z* 385.2/239.2 and *m/z* 385.2/112.0 (AMP 1018), *m/z* 418.1/104.1 and *m/z* 418.1/175.1 (Methionine-1018). This method was shown to be reliable and efficient for the identification and quantification of scorpion AMPs Kn2-7 and its D-isomer dKn2-7 in human serum samples by monitoring the transitions at *m/z* 558.7/120.2 and *m/z* 558.7/129.1 (Kn2-7/dKn2-7).

## Lay abstract

The rise of multidrug resistant (MDR) infections is a growing concern. Antimicrobial peptides (AMPs) have been found to be a promising new adjunct in the development of improved antimicrobial treatments to address this rise in MDR infections. The challenge of detecting and quantifying AMPs in biological matrices is a major limitation in fully developing this novel approach. Our work is a major step forward in providing a protocol for researchers who study a wide range of AMPs for many applications, in addition to the study of novel treatments for MDR infections.

The rapidly increasing threat of antibiotic-resistant infections has rendered an urgent need for the development of novel antimicrobial therapeutics. Antimicrobial peptides (AMPs) are oligopeptides with broad-spectrum activity against a number of pathogenic organisms such as viruses, bacteria, fungi and parasites [1]. In most cases, AMP immunodetection is challenging due to their low immunogenicity and small size. Recent advances in liquid chromatography–mass spectrometry (LC–MS) techniques have been enormously useful in peptide analysis. However, reports on the application of LC–MS for quantitative analysis of AMPs in complex biological samples are few [2]. This is due in part to the amphipathic nature of AMPs, which makes routine extraction and LC–MS analysis challenging. Therefore, the development of a protocol that permits rapid and accurate quantification of AMPs in biological samples is needed. Such a protocol is also essential for quality control and determination of batch-to-batch consistency in the study of potential AMP-based antimicrobial therapeutics. Herein, we show the optimization of a protocol for the extraction of AMPs from biological samples and a novel LC–MS method for the identification and quantification of AMPs (Supplementary Protocol).

## Materials & methods

Overnight BL21 culture is inoculated to 2 ml fresh LB media. It is incubated at 37°C at 350 rpm until O.D._600_ reaches 0.7–1.0, and inoculated with wild-type phage isolate at multiplicity of infection of 2.0 to lyse the bacteria, shaken in a 37°C incubator for 30 min and then the bacteria/phage cultures are harvested. Various organic solvents (methanol, ethanol, acetonitrile [ACN]) and acids (formic acid [FA], acetic acid, trichloroacetic acid [TCA]) [3–7] were evaluated for their efficiencies in AMP extraction from bacterial/phage cultures spiked with synthetic AMPs 1018 (VRLIVAVRIWRR) and methionine-1018 (Met-1018). By targeting both Gram-negative (e.g., *Pseudomonas aeruginosa* and *Escherichia coli*) and Gram-positive (e.g., *Staphylococcus aureus*) bacteria, AMP 1018 acts as a potent antibacterial: it kills bacteria, disperses biofilms and inhibits bacterial swarming [8]. Extracted AMPs were further purified and fractionated using 1cc Oasis HLB extraction cartridges (Waters, MA, USA).

**Figure 1.**
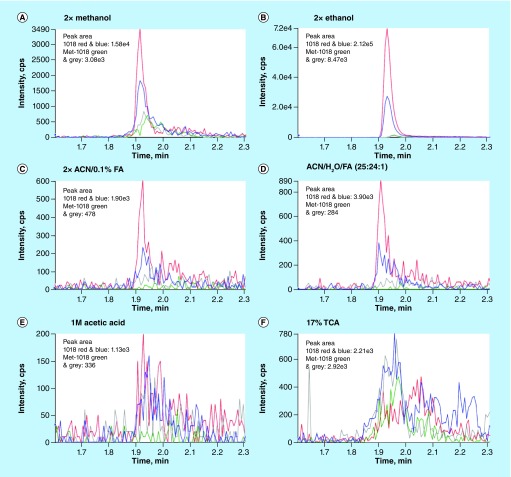
**Multiple reaction monitoring chromatograms of extracted and purified antimicrobial peptides 1018 and Met-1018 in bacterial/phage cultures.** Various solvents and acids were used for peptide extraction method development: 2× volumes of methanol **(A)**, 2× volumes of ethanol **(B)**, 2× volumes of ACN/0.1% FA **(C)**, ACN/H_2_O/FA (25:24:1) **(D)**, 1M acetic acid **(E)** or 17% TCA **(F)**. Peak area quantification data demonstrate that ethanol is a significantly more efficient solvent than the others for the extraction of AMPs 1018 and Met-1018 in bacterial/phage culture. ACN: Acetonitrile; FA: Formic acid.

Development of peptide extraction method for 1 μg/ml 1018 and 1 μg/ml Met-1018-spiked 2 ml bacterial/phage cultures was performed with three replicates with various protein precipitation reagents: 2× volumes of methanol, 2× volumes of ethanol, 2× volumes of ACN/0.1% FA, ACN/H_2_O/FA (25:24:1) and 1 M acetic acid or 17% TCA (Figure 1). After vortexing for 1 min, incubation on ice for 30 min and centrifuging at 17,000 × *g* for 20 min at 4°C, supernatants were collected and concentrated for further purification with an Oasis HLB cartridge before LC–MS analysis.

Our Oasis HLB cartridge fractionation procedure was performed as follows. The cartridge was equilibrated by washing sequentially with 1 ml 100% ACN, 1 ml 50% ACN and then 3 × 1 ml volumes of 5% ACN/0.1% FA. Collected peptide supernatants were loaded onto the cartridge. A light vacuum was applied to maintain the flow rate of 10 s/drop if the samples adhered to the cartridge. The cartridge was slowly (10 s/drop) washed with 4 × 1 ml volumes of 5% ACN/0.1% FA and then 2 × 1 ml volumes of 8% ACN/0.1% FA. The resultant 1018 and Met-1018 were eluted using 2 × 250 μl volumes of 17% ACN/0.1% FA and collected. Based on the initial LC–MS analysis of 1018 and Met-1018 with sequential elutions, 1018 and Met-1018 was eluted from Oasis HLB cartridge with 17% ACN/0.1% FA. Eluates were concentrated and transferred to 200 μl glass vials and an aliquot of 10 μl was injected for LC–MS analysis.

## Results & discussion

LC–MS analyses were performed using a two-component system composed of mobile phase A (10% ACN/0.1% FA in water) and mobile phase B (0.1% FA in 100% ACN) at a flow rate of 0.4 ml/min. Peptides were bound to an Agilent ZORBAX C18 column at 40°C for 0.5 min with 100% mobile phase A, and then were eluted with a linear gradient from 0 to 90% mobile phase B for 1.5 min. An API 4000 triple quadrupole MS was operated in multiple reaction monitoring (MRM) mode [9–11] via the positive electrospray ionization interface using two transitions for each analyte: *m/z* 385.2/239.2 and *m/z* 385.2/112.0 for 1018; *m/z* 418.1/104.1 and *m/z* 418.1/175.1 for Met-1018 (Figure 1). The MS/MS settings were: collision energy 39.9 V and collision cell exit potential 14.55 V for *m/z* 385.2/239.2; collision energy 43.0 V and collision cell exit potential 6.0 V for *m/z* 385.2/112.0; collision energy 30.0 V and collision cell exit potential 19.14 V for *m/z* 418.1/104.1; collision energy 31.0 V and collision cell exit potential 9.85 V for *m/z* 418.1/175.1. Capillary voltage was set at 4.5 kV. The data in Figure 1 demonstrate that ethanol was the most efficient organic solvent for the extraction of 1018 and Met-1018 from the biological samples. Met-1018 and 1018 in the 4^+^ charged ion form were found in the highest abundance in LC–MS compared with the 2^+^ and 3^+^ charged ion forms. The detection limit for 1018 and Met-1018 identification is 1.0 ng/l in our optimized LC–MS instrument.

**Figure 2.**
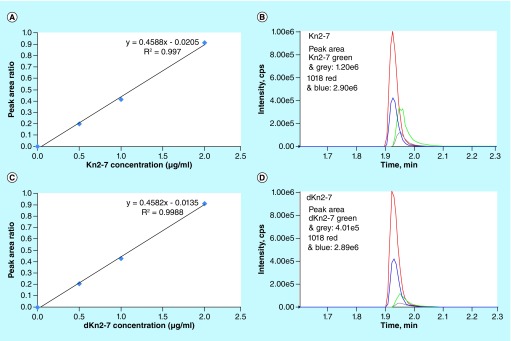
**LC–MS quantification of scorpion AMPs Kn2-7 and dKn2-7 spiked in human serum with our optimized protocol.** Calibration curves were constructed for Kn2-7 **(A)** and dKn2-7 **(C)**. MRM chromatograms of extracted Kn2-7 **(B)** and dKn2-7 **(D)** in human serum, as well as spiked internal standard AMP 1018, are shown.

Our optimized method was verified by applying the same approach to human serum samples spiked with 50 μg/ml scorpion AMPs Kn2-7 (FIKRIARLLRKIF) or its D-isomer (dKn2-7). Kn2-7 showed inhibitory activity against both Gram-positive bacteria and Gram-negative bacteria. Moreover, Kn2-7 exhibited antibacterial activity against clinical antibiotic-resistant strains such as methicillin-resistant *Staphylococcus aureus* [12]. 1 ml of Roswell Park Memorial Institute medium with 165 mM MOPS, pH 7.0, supplemented with 25% human serum was aliquoted into 1.5 ml Eppendorf tubes. Kn2-7 or dKn2-7 was added to yield a final concentration of 50 μg/ml. A 100 μl of solution was transferred to 200 μl of 100% ethanol. These samples were then stored at 4°C for 30 min and were subsequently centrifuged at 17,000 × *g* for 20 min. The supernatants were transferred to 500 μl Eppendorf tubes, then diluted eight-times with 5% ACN/0.1% FA and spiked with internal standard (IS) AMP 1018 (1 μg/ml). Spiked plasma standards at four concentrations over the range 0–2 μg/ml were prepared and analyzed in LC–MS runs. The calibration plots were constructed by weighted (1/x^2^) least-squares linear regression analysis of observed peptide-to-IS peak-area ratios against concentration. Extracted Kn2-7and dKn2-7 in spiked serum samples were quantified with two MRM transitions *m/z* 558.7/120.2 and *m/z* 558.7/129.1 by LC–MS analysis (Figure 2). Kn2-7 and dKn2-7 in samples were quantified as 22.78 and 7.97 μg/ml, respectively. The MS/MS settings were: collision energy 47.07 V and collision cell exit potential 17.07 V for *m/z* 558.7/120.2; and collision energy 61.01 V and collision cell exit potential 11.93 V for *m/z* 558.7/129.1. Capillary voltage was set at 4.5 kV.

## Conclusion

This novel methodology for extracting, identifying and quantifying AMPs in complex biological samples provides an effective and efficient means to discover new AMPs in both prokaryotes and eukaryotes for treating multidrug resistant bacterial infections and for other applications.

## Future perspective

Some AMPs are unstable in biological fluids, so protease inhibitors can be added to biological samples before peptide extraction to prevent their degradation. As techniques evolve, a more advanced LC–MS instrument could be used with this method to improve sensitivity and obtain lower limits of detection and quantification. We believe our optimized method could be applied to novel and general AMP identification and quantification in all kinds of biological matrices such as plasma and serum. Future investigations into potentially therapeutically active peptides will necessitate pharmacokinetic studies in animal models.

**Summary points**

- Ethanol was found to be the best solvent for antimicrobial peptide (AMP) extraction in biological matrices compared with other tested organic solvents and acids.
- An optimized peptide extraction and liquid chromatography–mass spectrometry protocol for identification and quantification of AMPs is described.
- This novel methodology is successful in extracting, identifying and quantifying amphipathic AMPs in complex biological samples.

## References

[1] Pushpanathan M, Gunasekaran P, Rajendhran J (2013). Antimicrobial peptides: versatile biological properties. *Int. J. Pept.*.

[2] Zhang RW, Liu WT, Geng LL, Chen XH, Bi KS (2011). Quantitative analysis of a novel antimicrobial peptide in rat plasma by ultra performance liquid chromatography-tandem mass spectrometry. *J. Pharm. Anal.*.

[3] Lutsiak ME, Kwon GS, Samuel J (2002). Analysis of peptide and lipopeptide content in liposomes. *J. Pharm. Pharm. Sci.*.

[4] Chertov O, Biragyn A, Kwak LW (2004). Organic solvent extraction of proteins and peptides from serum as an effective sample preparation for detection and identification of biomarkers by mass spectrometry. *Proteomics*.

[5] Taylor TM, Davidson PM, Zhong Q (2007). Extraction of nisin from a 2.5% commercial nisin product using methanol and ethanol solutions. *J. Food Prot.*.

[6] Kumar R, Thomas CM, Yong QC, Chen W, Baker KM (2012). The intracrine renin-angiotensin system. *Clin. Sci. (Lond)*.

[7] Mahatmanto T, Poth AG, Mylne JS, Craik DJ (2014). A comparative study of extraction methods reveals preferred solvents for cystine knot peptide isolation from Momordica cochinchinensis seeds. *Fitoterapia*.

[8] Mansour SC, de la Fuente-Nunez C, Hancock RE (2015). Peptide IDR-1018: modulating the immune system and targeting bacterial biofilms to treat antibiotic-resistant bacterial infections. *J. Pept. Sci.*.

[9] Escobar H, Kushnir MM, Rockwood AL, Meikle AW (2016). High sensitivity measurement of pancreatic polypeptide and its variant in serum and plasma by LC–MS/MS. *Methods Mol. Biol.*.

[10] De Marchi T, Kuhn E, Carr SA, Umar A (2015). Antibody-based capture of target peptides in multiple reaction monitoring experiments. *Methods Mol. Biol.*.

[11] Gillette MA, Carr SA (2013). Quantitative analysis of peptides and proteins in biomedicine by targeted mass spectrometry. *Nat. Methods*.

[12] Cao L, Dai C, Li Z (2012). Antibacterial activity and mechanism of a scorpion venom peptide derivative in vitro and in vivo. *PLoS ONE*.

