# Supplementary material for: Optimization of a peptide extraction and LC–MS protocol for quantitative analysis of antimicrobial peptides
Source: Future Sci OA. 2018 Oct 17;5(1):FSO348. doi: 10.4155/fsoa-2018-0073 (PMC6331757; doi:10.4155/fsoa-2018-0073)
Supplement: Supplementary file 1 [file fsoa-05-348-s1.docx]

**Peptide Extraction and LC-MS Protocol for Analysis of Antimicrobial Peptides in Biological Samples**

PROTOCOL FOR:

**Optimization of a Peptide Extraction and LC-MS Protocol for Quantitative Analysis of Antimicrobial Peptides**

*Future Science OA 0410 FSO348*

**REAGENTS**

Antimicrobial peptide (AMP) 1018 (VRLIVAVRIWRR) of 98% purity was synthesized in Genemed Synthesis, San Antonio, TX, USA.

Methionine-1018 of 98% purity, scorpion venom AMP Kn2-7 (FIKRIARLLRKIF) and its D-type-amino-acid isomer dKn2-7 of 96% purities were synthesized in GenScript, Piscataway, NJ, USA.

Acetonitrile (ACN), Ethanol, Formic acid (FA), HPLC grade H_2_O (Sigma-Aldrich, St. Louis, MO, USA)

ZORBAX Eclipse XDB 80Å C18 column (2.1 x 50 mm, 5 µm) (Agilent Technologies, Santa Clara, CA, USA)

Oasis HLB 1cc Vac Cartridge, 30mg Sorbent per Cartridge, 30 µm Particle Size (Waters, Milford, MA, USA)

**PROCEDURE**

AMPs peptide extraction with ethanol solvent and AMPs peptide purification with an Oasis HLB extraction cartridge for, for example, 1 µg/mL AMPs 1018/Methionine-1018 spiked bacterial/phage cultures.

1. Add 2× volumes of -20°C ethanol to each biological matrix (culture media, serum, saliva, plasma, etc.) for protein precipitation and peptides extraction, immediately vortex for 1 min and incubate on ice for 30 min.
2. Centrifuge at 17,000× g for 20 min at 4°C. Insoluble cellular debris from the precipitation reaction is removed. Collect 6 mL of supernatant and concentrate to 0.5 mL using a SpeedVac concentrator.
3. Activate Oasis HLB extraction cartridges with 1 mL 100% ACN and then 1 mL 50% ACN/0.1% FA in water, and equilibrated with 3× 1 mL 0.1% FA in water.
4. Concentrated peptides from step 2 are diluted 4× volumes with 5% ACN/0.1% FA, and then loaded onto the equilibrated HLB cartridge for desalting and purification.

***HINT:*** *A light vacuum may be applied to the cartridge to maintain the flow rate of 10 sec/drop during cartridge loading, washing and eluting in case the solution adheres to the cartridge.*

1. Wash the cartridge with 4× 1 mL of 5% ACN/0.1% FA and then 2× 1 mL of 8% ACN/0.1% FA.
2. Elute peptides from the cartridge with 2× 250 µl of 17% ACN/0.1% FA and collect eluate in a polypropylene tube. Dry 500 µl eluate using a SpeedVac concentrator, then reconstitute in 40 µl 5% ACN/0.2% FA.

***ATTENTION:*** *0.2% FA instead of 0.1% FA is used in the reconstituted solution to ensure data linearity by fully protonating highly basic AMPs and avoiding their ionized variability before LC-MS analysis.*

***REST:*** *Reconstituted peptide samples can be stored at -20* ºC *for up to one week before LC-MS analysis.*

1. Transfer reconstituted eluate to 200 µl glass vials. Inject 10 µl aliquot for LC-MS analysis.

LC-MS analysis of extracted and purified AMPs

1. A gradient elution is carried out on an Agilent ZORBAX Eclipse XDB 80Å C18 column (2.1 x 50 mm, 5 µm) at 40°C with mobile phase A (10% ACN/0.1% FA in water) and mobile phase B (100% ACN/0.1% FA). Peptides are bound to the column for 0.5 min with 100% mobile phase A, and then are eluted with a linear gradient from 0 to 90% mobile phase B for 1.5 min. Between 2 and 3.5 min, the mobile phase are maintained at 90% B. From 3.5 to 6 min, the mobile phase is switched back to 100% A. The total flow rate of mobile phase is 0.4 ml/min. Under this chromatographic condition, the retention time for AMPs 1018, Methionine-1018 and Kn2-7/dKn2-7 were 1.93 min, 1.94 min, and 1.95 min, respectively.
2. MS/MS measurements are performed in the positive electrospray tandem MS mode, i.e., monitoring the transitions at *m/z* 385.2/239.2 and *m/z* 385.2/112.0 (AMP 1018), *m/z* 418.1/104.1 and *m/z* 418.1/175.1 (Methionine-1018), *m/z* 558.7/120.2 and *m/z* 558.7/129.1 (Kn2-7 and dKn2-7). The MS/MS settings are: declustering potential 73.7 V, collision energy 39.9 V and collision cell exit potential 14.55 V for *m/z* 385.2/239.2; declustering potential 73.7 V, collision energy 43.0 V and collision cell exit potential 6.0 V for *m/z* 385.2/112.0; declustering potential 78.5 V, collision energy 30 V and collision cell exit potential 19.14 V for *m/z* 418.1/104.1; declustering potential 78.5 V, collision energy 31.0 V and collision cell exit potential 9.85 V for *m/z* 418.1/175.1; declustering potential 87.83 V, collision energy 47.07 V and collision cell exit potential 17.07 V for *m/z* 558.7/120.2; declustering potential 87.83 V, collision energy 61.01 V and collision cell exit potential 11.93 V for *m/z* 558.7/129.1. Capillary voltage was set at 4.5 kV.

***ATTENTION:*** *Due to these AMPs’ multiple basic amino acids composition, AMPs’ quadruply charged ions [M+4H]^4+^ and triply charged ions [M+3H]^3+^ are found most intensively in the full scan ESI mass spectra, while their quintuply and doubly charged ions are rarely detected.*

**EQUIPMENT**

Centrifuge 5430 R (Eppendorf, Hamburg, Germany)

SAVANT SPD131DDA SpeedVac Concentrator (Thermo Fisher Scientific, Waltham, MA, USA)

Shimadzu HPLC system LC-20AD (Shimadzu Corporation, Kyoto, Japan)

AB Sciex API 4000 Triple Quad mass spectrometer (SCIEX, Framingham, MA, USA)
